# Assessing food security among young farmers in Africa: evidence from Kenya, Nigeria, and Uganda

**DOI:** 10.1186/s40100-023-00246-x

**Published:** 2023-02-23

**Authors:** Dolapo Adeyanju, John Mburu, Wainaina Gituro, Chepchumba Chumo, Djana Mignouna, Adebayo Ogunniyi, John Kehinde Akomolafe, Joseph Ejima

**Affiliations:** 1 Department of Agricultural Economics, University of Nairobi, Nairobi, Kenya; 2 Department of Management Science, School of Business, University of Nairobi, Nairobi, Kenya; 3 International Institute of Tropical Agriculture (IITA), Ibadan, Nigeria; 4 International Fund for Agricultural Development (IFAD), Abuja, Nigeria; 5 Department of Economics, Afe Babalola University, Ado-Ekiti, Ekiti State Nigeria; 6 International Food Policy Research Institute, Abuja, Nigeria

**Keywords:** Food consumption, Food security, Young farmers, Africa, Determinants

## Abstract

Food insecurity remains a serious challenge for many households in Africa and the situation is even more prevalent among young people. However, there is a dearth of empirical evidence on youth food security status in Africa. We assessed the level and determinants of food security among young farmers in Africa. We adopted a multi-stage sampling technique to select 400, 429, and 606 young farmers in Kenya, Nigeria, and Uganda, respectively. Individual food consumption was assessed following a 7 days recall method. The Food Consumption Score, which combines dietary diversity and consumption frequency was used to assess food security status while the determinants of food security were identified using a logistic regression model. Results suggest low dietary diversity across the three countries. Also, the majority of the respondents had an unacceptable food consumption score, suggesting that despite being food producers, young farmers are still food insecure. The odds of being food secure was positively determined by access to extension services, participation in the ENABLE TAAT business incubation programme, and access to market information but, negatively by access to credit, number of employees, Covid-19 pandemic, and location. Additionally, the food security status of young female farmers was positively influenced by age, suggesting that younger youths are less food secure compared to older ones. These results suggest that more efforts should be directed towards improving the food security of young African farmers and that policy- and programme-level interventions should support access to extension services, market information, and land. Additionally, more investments should be directed towards developing need-based agribusiness incubation programmes with an effort to scale existing programmes beyond the regular one-time period.

## Introduction

One of the United Nations' (UN) Sustainable Development Goals (SDGs) is to end hunger and ensure food accessibility by all people by 2030. While there have been several national and international efforts to achieve this goal, food insecurity remains a major livelihood challenge in many developing countries, particularly in Africa. A recent report by FAO (2021) indicates that about 21% of the African population faced severe hunger in 2020, doubling the proportion reported by other regions in the World. Also, over 50% of the population faces moderate to severe food insecurity (Gebre 2021; World Health Organization, 2019), representing one-third (799 million) of the estimated 2.37 billion people facing moderate to severe food insecurity in the World (FAO 2021). Compared to other regions, the prevalence of food insecurity is much higher in West and East Africa, where over 68% and 65%, respectively experience moderate to severe food insecurity (FAO 2021).

While food security has received significant attention in the empirical literature in recent years due to the 2030 UN agenda (Masa et al. 2020), existing studies seem to be skewed toward the household, with scanty evidence on young adults. According to Sithole and Dinbabo (2016), youths, defined as people between 18 and 35 years old, belong to a vulnerable group subjected to high rates of unemployment, social segregation, stigmatization, and low incomes, which affect their livelihoods and economic status. Similarly, Hadley et al. (2009) described youth as a forgotten population experiencing a range of social, biological, and mental transitions as they shift into adult roles. Among other livelihood issues, living without food is a serious issue that disproportionately affects young Africans (Masa et al. 2020), who account for over 60% of the African population (African Development Bank et al. 2017). This corroborates Elgar et al. (2021) who posit that youth face moderate to severe food insecurity and other livelihoods, economic, and health risks resulting from the negative implications of severe food insecurity.

It is worth noting that based on the relevance of agriculture to rural economies and, consequently, the livelihood of youths, concerted efforts are being made by both national and international stakeholders to promote agricultural employment among rural youths in Africa (Adeyanju et al. 2021a, b; Yami et al. 2019). Most of these efforts are being implemented to curb the rising rate of youth unemployment and ensure that rural youths engage in economic activities for better livelihood outcomes. For instance, the ENABLE-TAAT programme implemented by the International Institute of Tropical Agriculture (IITA) under the funding of the African Development Bank (AfDB) aims to help young farmers develop relevant agricultural and business skills to improve their livelihood and food security status. Thus, within this context, where strategies and efforts are ongoing to improve youth livelihoods through agriculture (Adeyanju et al. 2021a, b; Yami et al. 2019), it is important to assess their food consumption to ensure that their food security is also being addressed. This is because, compared to other categories of the population, food insecurity can be especially hard on young people maturing from adolescence into “emerging adulthood” (Larson et al. 2020). This is supported by Acheampong et al. (2022) who posit that farmers in rural areas experience heightened food insecurity and hunger despite growing and marketing food crops.

Though studies have been conducted on youth food security in Africa, the bulk of available pieces of literature focus mostly on children, university students, and other socially vulnerable groups (Davidson and Morrell 2020; Larson et al. 2020; Leung et al. 2019; Loftus et al. 2021; McArthur et al. 2018; Ragasa et al. 2019; Tong et al. 2019) while studies on the food security status of young farmers are quite limited. The evidence that has been provided so far does not give grounds for adequate policy suggestions on young African farmers. This is because studies that considered young farmers across Africa are quite hard to find. Specifically, there is very limited peer-reviewed research on the level and determinants of food security among young farmers in developing countries. This highlights a critical evidence gap in a region where youths are more likely than adults to lack timely access to adequate food (Masa et al. 2020). Thus, it is imperative to empirically understand the depth and determinants of food security among young farmers from a demographic perspective to ensure that they are continuously being captured in food security debates. Furthermore, this evidence is important to dissociate youths enrolled in schooling from those engaged in economic activities since the latter may be less dependent on family members for their basic needs (Masa et al. 2020). Also, this evidence will inform timely and appropriate targeting of vulnerable youths and identify the unique factors to target to reduce food insecurity among young people.

This study aims to generate empirical evidence that could guide policymakers and relevant stakeholders in addressing food insecurity among young farmers in Africa. Specifically, the study seeks to answer the following questions: 1. Do young farmers in Africa face food insecurity? 2. What are the determinants of food security among young African farmers? Also, the study was interested in gaining new insight into youths' food choices and sources while comparing these with the household consumption pattern found in other studies. For better insights, the result was disaggregated by gender to assess the common and different factors that influence food security among young male and female farmers. In answering these questions, the study adopted the World Food Programme’s (WFP) Food Consumption Score (FCS) based on the consumption frequency of specific food groups weighted based on their respective nutritional importance. Also, the determinants of food security were identified using a logistic regression model. The results show high consumption of starchy and fatty/oily foods against proteinous foods during the 7-day recall period, suggesting low dietary diversity which could be a result of high reliance on own production and rising food prices. Also, despite their engagement in food production, the results show that food insecurity was high among the youths, with the highest prevalence in Uganda. Among other factors, participation in agribusiness programmes, access to extension services, credit, and market information was relevant to food security. This implies that efforts to improve the food security status of young farmers must consider these factors for better outcomes. These results have clear implications for youth food security debates and policy, especially during this period of multi-faceted economic crisis and youth bulge.

### Conceptualizing food security

Within the context of this study, food security exists “when all people, at all times, have physical, social, and economic access to sufficient, safe, and nutritious food that meet their dietary needs and food preferences for an active and healthy life” (FAO 2009, p. 1). This definition identifies four major dimensions of food security including availability, access, affordability, and utilization. Food availability denotes having sufficient food in both formal and informal markets which meet both individual and market demand. This also entails raising food production locally and through imports. Food accessibility is having sufficient resources (both economic and physical) to acquire sufficient foods needed for a nutritious diet (Acheampong et al. 2022). This entails an individual’s ability to obtain their food need on a timely and regular basis. In the case of smallholder farmers, food access is mostly facilitated through their own production, gifts, exchange, borrowing, food aid, and purchase. Evidence abounds that the majority of smallholder farmers largely depend on their own production which could limit their access to other food items they don’t produce (Huang and Tian 2019; Nchanji and Lutomia 2021). Food utilization entails how individuals or household use the food items available and accessible to them while food stability encompassed the other three dimensions, looking at steady availability, accessibility, and utilization of food at all times (Acheampong et al. 2022). According to van Meijl et al. (2020), it is not just the quantity of food available, but also the ability to access it physically and financially that matters, along with the ability to utilize food well through good processing and preparation practices and the stability of these three components at all times.

While this definition of food security has been widely accepted, defining a common metric to identify the common factors influencing food insecurity across different countries, population categories, and age groups is lacking (Smith et al. 2017). This is because the root causes of food insecurity are multidimensional and difficult to understand, particularly in Africa (Kansiime et al. 2021), thereby raising the need to understand the depth and determinants of food (in)security across different ages and population categories (Wieck et al. 2014). According to Matavel et al. (2022), a critical aspect of strategies to achieve food security is the identification of food-insecure individuals or households and the characterization of the nature of their insecurity through measurements which will in turn provide a basis for monitoring the progress and impact of food security efforts. The current study aims to fill this research gap by assessing the food security status and identifying factors affecting food security among young African farmers. In achieveing this, the WFP’s FCS was adopted to measure food security based on its ability to accurately capture dietary diversity which is important to facilitate policy debates on youth food security.

### Determinants of food security among young farmers in Africa

According to Masa et al. (2020), the research gap on youth food insecurity in Africa could be attributed to several factors, including the lack of suitable data and validated measures. While economic factors such as farm production and income are commonly linked to food security (Mulwa and Kabubo-Mariara 2022), this approach has been criticized since income is highly variable for those engaged in farming activities, suggesting a need to capture other relevant correlates such as socioeconomic, demographic, and institutional factors to inform effective targeting and practical policy on youth food security. For instance, some studies have found a significant link between food security and gender, social capital, and empowerment programmes (Devereux et al. 2020; George et al. 2020; Larson et al. 2019; Sseguya et al. 2018). According to Masa et al. (2020), the heterogeneity of the youth population depicts varying economic, physical, and social characteristics which could invariably influence their food security status in ways that differ from adults and other vulnerable groups. The authors argued that while youth in the lower age category (aged 15–18 years) may remain dependent on their parents or family members for food, the older ones are more likely to be less dependent, suggesting the need to examine the relevant factors influencing food security among this category. Also, in identifying these factors, it is equally important to consider their influence from a gender perspective since food allocation may be biased against young women, which could mean that they receive smaller portions or a less diverse diet (Aurino 2017). In essence, young women may be at risk of severe food insecurity compared to their male counterparts. This is supported by Harris-Fry et al. (2018) who attributed the gender bias in food allocation to varying degrees of resources at an individual or household’s disposal. Thus, in addition to identifying factors that influence food security among young farmers, we assessed whether significant socioeconomic correlates of food security varied by gender.

## Materials and methods

### Data

This study uses the Youth in Agriculture Survey (YAS) data collected under the ‘ENABLE-TAAT’ project^1^ funded by the African Development Bank (AfBD) and facilitated by the International Institute of Tropical Agriculture (IITA). The survey was conducted in Kenya, Nigeria, and Uganda between August and December 2021. The study adopted a cross-national sampling design following Kaminska and Lynn (2017). Thus, different sampling frames were used in selecting the respondents in each country. In essence, samples were independently selected in each country, using a single stratum indicator that reflects the same sampling strata within each country.

To obtain a random sample, a multi-stage stratified random sampling technique was adopted in selecting the respondents. The first stage involves the purposive selection of three countries out of the seven countries in which the ENABLE-TAAT programme was conducted in 2018. The choice of these countries was based on the high number of young farmers who participated in the programme compared to the other countries in which the programme was implemented in the pilot year (2018), their ranks in the level of youth unemployment and poverty among the project countries, and the advanced level of stakeholder engagement with IITA in youth agribusiness development in Africa. The second stage involves randomly selecting youths from three sampling frames to make a sample size of 1463, following Yamane (1967). The sampling frame for each country consisted of a complete list of young farmers registered in the database of the ENABLE-TAAT programme in the reference year which included 744, 805, and 1119 young farmers in Kenya, Nigeria, and Uganda, respectively. The sample size for each country was determined based on probability proportional to size giving a sample size of 408, 441, and 614 for Kenya, Nigeria, and Uganda, respectively. Finally, the random selection of the respondents was done using random numbers generated via Microsoft Excel.

A total of 1435 young farmers fully participated in the survey across the three countries. Out of this, responses were obtained from 400, 429, and 606 respondents in Kenya, Nigeria, and Uganda, respectively, representing a 98% response rate which is sufficient for the analysis. The missing individuals (2%) from the survey data were due to individual-level refusals to complete the survey. The selected youths were interviewed face-to-face by trained enumerators with a well-structured questionnaire programmed on Open Data Kit (ODK), which was carefully designed and pre-tested before the main survey. In addition to demographic and farm-level data, data on various food items, sources, and consumption patterns were collected. Food consumption was captured through a 7-days recall recommended by WFP. Specifically, the youths were asked how frequently (in days) they consumed specified food items in the last 7 days preceding the survey. Also, respondents were requested to indicate their current residence, and results showed that responses were skewed towards rural areas across the three countries. This further indicates the importance of agriculture to rural youths (Cousins et al. 2018; Sithole and Dinbabo 2016).

### Measurement of food consumption score (FCS)

The FCS also referred to as a “food frequency indicator,” is a frequency-weighted diet diversity score calculated using the consumption frequency of eight food groups, including main staples, pulses, vegetables, fruit, meat and fish, milk, sugar, and oil, over a 7-days recall period (Wiesmann et al. 2009). It indicates the dietary diversity, consumption frequency, and sources of these food items. In this study, the FCS was constructed using the information on food consumption gathered from a country-specific list of food items. The relevance of these food items to food security has been widely discussed in the literature. While some food items such as cereal grains are common staples that are easily accessible and affordable by many Africans (Brouns et al. 2019; Fukagawa and Ziska 2019; Poole et al. 2021), other items such as exotic fruits and dairy products are quite expensive and rarely found in African diet. For instance, Fukagawa and Ziska (2019) document that over 20% of the world's calories come from rice while cereal grains provide the world’s population with the most accessible and affordable macronutrients (energy and protein). However, sustainable food security cannot be realized by dependence on a few crops such as rice, maize, wheat, and soybeans that account for a major part of the global food supply (Aworh 2023).

In this study, respondents were asked how many days they had consumed different food items in the week before the survey. These food items were grouped into eight specific food categories, as presented in Table 1. The consumption frequencies of the eight groups were summed, and any frequency value above seven was capped at seven. Next, the frequency obtained for each food group was multiplied by an assigned weight (see Table 1) that is based on its nutrient content. Finally, the FCS was computed as the sum of the weighted value of all the food groups.

**Table 1:** Food groups and weight. *Source* United Nations World Food Programme (2008)

Food Items	Food groups	Weight
Maize, rice, pasta, bread, and other cereals	Cereals and Tubers	2
Cassava, Yam, Arrow roots/Cocoyam, and potatoes		
Vegetables and leaves	Vegetables	1
Fruits	Fruit	1
Beef, goat meat, poultry, pork, eggs, fish, other meat, and seafood	Animal protein	4
Beans, peas, lentils, peanuts, and others	Pulses	3
Milk and other milk products	Milk	4
Sugar, honey, and sugar products	Sugar	0.5
Edible oils, fats, and butter	Oil	0.5

This method of assessing food consumption has been adopted to compute individual and household food consumption by many studies in developing countries (Aweke et al. 2021; de Menezes-Júnior et al. 2022; Fite et al. 2022). The formula used in computing the food consumption score is presented as:

1 $${\text{FCS}} = \Sigma F_{i} X_{i}$$

where $F_{i}$. represents the different food groups, and $i$. is the different food items. $X_{i}$ denotes the consumption frequency of each food group over the past seven days. Finally, the continuous FCS was categorized into appropriate thresholds of food consumption groups as follows: poor food consumption (FCS = 0–28), borderline (FCS = 28.5–42), and Acceptable food consumption (FCS > 42) following United Nations World Food Program (2008).

### Empirical model

One of this study’s objectives is to identify factors that determine food security among rural young farmers in Africa. Thus, the primary focus is on assessing whether a young farmer has an acceptable FCS or not. On this note, the FCS was re-grouped into two, with the poor and borderline categories deemed unacceptable. As a result, the outcome variable, FSC, is a dichotomous one, classified as acceptable (assigned a numeric value of 1) and unacceptable (assigned a numeric value of 0). Based on this dichotomy nature of the outcome variable, the determinants of food consumption were identified using a logistic regression model presented in Eq. (2):

2 $$\begin{aligned} & y_{i} = \alpha_{i} x_{i} + e_{i} \\ & {\text{where }}\,\,y_{i} = \left\{ {\begin{array}{*{20}l} {1\, = \,{\text{Acceptable}}\,{\text{ FCS}}} \hfill \\ {0\, = \,{\text{Unacceptable}} \,{\text{FCS}}} \hfill \\ \end{array} } \right. \\ \end{aligned}$$

The outcome is believed to be influenced by a vector of explanatory variables $x_{i}$; $\alpha_{i}$. denotes a vector of parameters to be estimated; and $e_{i}$. is the error term.

The assumption is that the probability of $y_{i}$. assuming the value of 0 and 1 is $1 - P_{i} {\text{and}} P_{i}$. respectively.where

3 $$P_{i} = \frac{{e^{y} }}{{1 + e^{y} }}$$

The marginal effects of explanatory variables can be eimated aspecified in Eq. (4):

4 $$\beta_{m.e.} = \left[ {{\raise0.7ex\hbox{${\partial \left( {\alpha_{i} x_{i} + e_{i} } \right)}$} \!\mathord{\left/ {\vphantom {{\partial \left( {\alpha_{i} x_{i} + e_{i} } \right)} {\partial \left( {\alpha_{i} x_{i} } \right)}}}\right.\kern-0pt} \!\lower0.7ex\hbox{${\partial \left( {\alpha_{i} x_{i} } \right)}$}}} \right]\beta_{i}$$

The choice of the variables included in the logit model was guided by relevant studies, including Davidson and Morrell (2020), Feleke et al. (2005); Ngema et al. (2018), Nkomoki et al. (2019), Tong et al. (2019), and Usman and Callo-Concha (2021). These variables, their measurements, and hypothesized direction are presented in Appendix 1.

### Descriptive results

The descriptive results of the variables included in the logit model are presented in Table 2. Generally, the average age of the respondents was about 29 years. Similar results were obtained for Kenya and Nigeria, while the mean age for the Uganda respondents was 27 years. This shows that most of the respondents belong to the older youth category (Gardner, et al. 2015) and hence, are in their economically productive years. Over half of the respondents are male, suggesting a gender balance among the respondents. Even though this was not pre-determined, it could be attributed to the promotion of women empowerment in many African countries and the deliberate focus of the ENABLE TAAT programme on more young women engaged in agriculture.

**Table 2:** Descriptive statistics for the socioeconomic and demographic characteristics of the respondents

Variable	Pooled	Kenya	Nigeria	Uganda
	*n* = 1435	*n* = 400	*n* = 429	*N* = 606
Age of respondents (Years)	28.50	29.04	29.59	27.38
Education (Years)	14.21	13.74	15.61	13.52
Household Size (#)	4.96	5.15	4.78	4.95
Land size (Ha)	2.14	2.34	1.88	2.18
Number of employees (#)	4.06	3.69	4.06	4.24
Number of young employees (#)	3.44	3.04	3.64	3.52
Farming experience (Years)	3.41	3.06	3.16	3.823
Value of productive assets (#)	1886.15	2183.74	1095.19	2249.65
Gender (Male)	57.35	55.50	66.90	51.82
Access to credit (%)	39.86	21.75	20.98	65.18
Access to extension services (%)	60.56	59.75	59.44	61.88
Access to market (%)	62.65	62.50	62.70	62.71
Residence (Rural)	73.59	94.00	32.87	88.94
Sole business ownership (%)	80.91	88.75	80.89	75.74
Access to training (%)	51.36	45.75	53.61	53.47
Asset ownership (%)	94.84	96.25	97.90	91.75
Part-time engagement in farming (%)	21.95	22.00	23.54	20.79
Affected by Covid-19 (%)	73.80	69.25	73.19	77.23

Source: Survey data (2021)

The respondents were quite learned, having about 14 years of formal education. By country, the respondents from Nigeria had two additional years of formal education than those from the other two countries. However, this could be attributed to the educational system of different countries. Overall, the results imply that a larger proportion of the respondents had at least tertiary education (between 14 and 16 years). This is not surprising since the respondents are from a list of young graduate farmers. The pooled-mean household size, defined by the number of persons that lives and dines with a respondent, was 5 persons. Other studies have shown that the average household size in Africa is between 5 and 7 persons (Makwinja et al. 2021; Nwosu et al. 2020; Omara et al. 2021). The result also indicates that respondents across the three countries had an average farmland of 2 hectares, suggesting that young farmers engaged in food production operate on small farmlands which could be a result of limited access to land. This is well documented in literature as one of the significant factors affecting the performance of youth-owned agro-enterprise (Adeyanju et al. 2021a, b; Ricker-Gilbert and Chamberlin 2018; Yeboah et al. 2020).

Also, the respondents across the three countries were quite similar regarding the number of hired employees, with an average of about 4 employees. Interestingly, the results show that three-quarters of the people hired were young people, indicating the preference of young farmers for young labours. This preference could be because they share common features and could relate better with their peers than adults. While this is not the focus of this study, the results suggest a trend in peer-to-peer employment which could have notable implications for reducing youth unemployment in Africa. Except Uganda, with more years of experience by 1 year, respondents from Kenya and Nigeria had about 3 years of farming experience. This suggests that they are operating within the growth stage of agribusiness and are likely to have a strong customer base. It also appears that the majority of the respondents started their agribusinesses after participating in the ENABLE TAAT programme, indicating the relevance of agribusiness empowerment programmes in stirring the intention of young graduates to engage in agriculture.

Access to credit was quite low among the respondents in Kenya and Nigeria (21.75% and 20.98%, respectively), which could be attributed to the lack of creditworthiness among young people (Buszko et al. 2020; Ndagijimana et al. 2018). However, contrary to many studies conducted in Uganda (Bukuluki et al. 2020; Mulume et al. 2022), more respondents in Uganda (65%) had access to credit. This could be because they had relatively more years of farming experience or could be as a result of the recent efforts by the Ugandan government to facilitate youth access to affordable credit (Gunewardena and Seck 2020). The majority of the respondents (60% and 63%, respectively) had access to extension services and markets. Differences were not found in individual countries regarding access to extension and markets. Compared to Nigeria, where only one-third of the respondents reside in rural areas, most of the respondents in Kenya and Uganda (94% and 90, respectively) are domiciled in rural areas, indicating some demographic similarities between the eastern countries. The result obtained for Nigeria is, however, not surprising considering the degree of rural–urban migration and the growth of innovative farming such as soilless farming in Nigeria in recent years (Olubanjo and Alade 2018; Ovharhe et al. 2020). With the exception of Kenya where less than half of the respondents had access to training, over half of the respondents from the other countries had accessed training in the last 12 months. Nearly all the respondents had productive assets worth an average of 1886 USD. This shows a considerable level of asset ownership which could be an important factor for food consumption. This is because higher value of asset could imply increased production and income which could be used to smoothen food consumption. Less than one-fourth (< 25%) of the respondents had other forms of employment, implying that the majority are solely dependent on agricultural employment. Engagement in other forms of employment implies that productive time is shared between different activities. The implication is that, while additional source of income could add to total income, the time allocated to agricultural activities is reduced, thereby reducing outputs and subsequently, farm income. Considering that most farmers consume own-produced food, reduced output could reduce food consumption.

Because of the recent Covid-19 pandemic, this study captured the effect of the pandemic on agricultural activities and performance. While the level and nature of the impact differ, nearly all the respondents stated that the Covid-19 pandemic affected their agricultural activities and performance through the measures adopted to curtail the pandemic. This is consistent with an emerging body of literature that has documented the impact of the Covid-19 pandemic on economic activities and food security (Amare et al. 2021; Ayanlade and Radeny 2020; Davila et al. 2021; Stephens et al. 2020; World Bank 2020).

## Results and discussions

### Young farmers’ dietary diversity

**Fig. 1:**
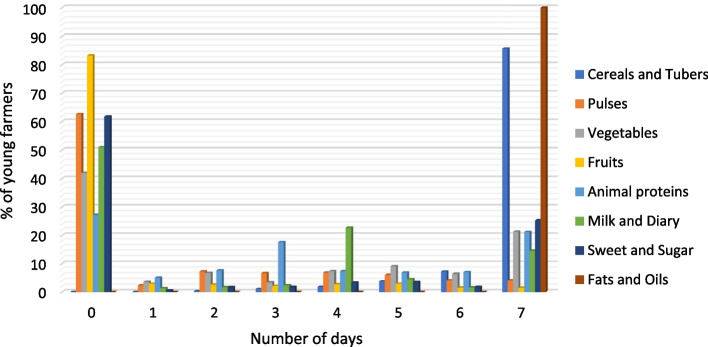
Youth consumption of different food groups over a 7-days recall period

Figure 1 shows that fats and oils were the most consumed among the respondents across the three countries. This corroborates Shim et al. (2021) who attribute the high consumption of fatty foods to cheap cost and minimal preparation time which often lead to overconsumption. Cereals and tubers were consumed every day by 86% in the 7-days recall period across the three countries. This could be because food items in this category are mostly farm-sourced and readily available through their production. The results also showed that pulses and fruits, respectively were the least-consumed food groups, by only 2% and 4% of the respondents during the 7-days recall period. This corroborates Workicho et al. (2016) who associated the low consumption of fruits among Ethiopian farmers with limited access to diet diversity markets and the associated cost of purchasing these items since smallholders’ production is centered around staples and livestock production. Similarly, Acheampong et al. (2022) found that the consumption of pulses and fruits was not as common as roots and tubers, and cereals among farming households in Ghana. Also, the results indicate that 42%, 27%, and 51% had no vegetables, animal proteins (Fish and Meat), and milk and dairy products during the recall period. The pattern shown in these Figures is consistent with common knowledge that individuals in Africa generally consume foods rich in carbohydrates and oils, with starches being more common than proteins. This raises a key concern that most African youths appear to be missing the macro and micro-nutrients present in proteinous foods and also questions their knowledge on dietary diversity.

**Fig. 2:**
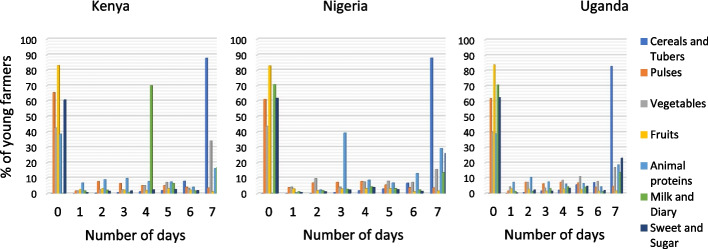
Food consumption in Kenya, Nigeria, Uganda

Data presented in Fig. 2 show a similar trend in the study countries, indicating that the African food system consists largely of cereals and root crops which forms a larger part of individual diets. Also, the consumption of fruits was very low in each of the countries. Milk consumption was relatively high in Kenya compared to the other countries, where all the respondents consumed milk and dairy products at least once in the 7-days recall period. This could be because milk production is high in Kenya compared to other countries. Consumption of animal protein was high in Nigeria where all the respondents equally reported consumption at least once in the recall period. These results suggest that food consumption among the respondents is skewed towards cereals and tubers and consumption of varied diets, which is a key approach to achieving nutritional requirements is poor.

### Food consumption score

Table 3 presents the average food consumption scores (FCS) and respondents’ profiles by country. Table 4 shows less than half (48%) of the youths across the three countries were within the acceptable FCS, suggesting that despite their engagement in food production, the majority of young farmers in Africa are still food insecure. Interestingly, different results were obtained in each country. While the majority of respondents in Kenya and Nigeria (61% and 51%, respectively) had acceptable FCS, only a little above one-third (38%) had acceptable FCS in Uganda. Although there was a substantial number (39%) of youths on the borderline FCS group across the countries, Nigeria had the highest number with 48%. Overall, food insecurity was highest among the respondents in Uganda corroborating other studies that have found low food consumption among young people in developing countries (Bhawra et al. 2021; Cady 2014). The relatively high FCS obtained among the respondents in Nigeria could be attributed to crop diversity and favorable climate conditions in terms of rainfall and temperature compared to the other countries. Farmers from Nigeria are more likely to have the luxury to produce diverse crops that contribute to food security. Even though the food consumption disparity between the two East African countries is surprising, it could be attributed to differences in food choices (for instance, high milk and dairy product consumption in Kenya) and Kenya being the hub of East Africa’s economic activities. Generally, despite being food producers, about 52% of the respondents across the three countries were found to be food insecure. This corroborates Masa et al. (2020) who found that more than half of African youths experience moderate to severe food insecurity and Acheampong et al. (2022) who found that despite being food producers and marketers, farmers still experience food insecurity in Africa.

**Table 3:** Category of food consumption score by country

Food consumption profile	Pooled	Kenya	Nigeria	Uganda
	*n* = 1435	*n* = 400	n = 429	*N* = 606
Average food consumption score (mean)	45.15	51.32	47.64	39.32
Poor Food consumption (%)	13.24	0.00	0.93	30.69
Borderline food consumption (%)	38.75	39.00	48.48	31.68
Acceptable food consumption (%)	48.01	61.00	50.58	37.62

*Source* Survey data (2021)

**Table 4:** Determinants of food consumption among the respondents

Variables	M.E	Std. Err	M.E	Std. Err	M.E	Std. Err
	Pooled = 1435		Male = 823		Female = 612	
Extension service	0.160***	0.080	0.150***	0.106	0.170***	0.124
Gender	0.004	0.071				
Credit	− 0.124***	0.087	− 0.138***	0.119	− 0.119***	0.131
(Log)Age	0.030	0.219	− 0.135	0.305	0.220**	0.323
Education	0.019	0.060	0.034	0.082	0.006	0.092
(Log)Household size	0.044	0.079	0.036	0.103	0.051	0.130
Residence	− 0.012	0.061	− 0.030	0.079	0.020	0.102
Ownership	0.040	0.090	0.030	0.131	0.051	0.128
ENABLE TAAT	0.105***	0.078	0.100***	0.104	0.103**	0.122
Employees	− 0.059**	0.075	− 0.026	0.099	− 0.113***	0.118
Market information	0.197***	0.079	0.198***	0.104	0.201***	0.125
Asset ownership	0.084	0.161	0.054	0.224	0.111	0.238
Covid19	− 0.071**	0.080	− 0.029	0.108	− 0.120***	0.123
Land size	0.002	0.015	− 0.004	0.020	0.013	0.027
(Log)Farm income	0.034	0.104	0.029	0.128	0.055	0.186
*Country dummies*						
Kenya	− 0.079***	0.047	− 0.100***	0.066	− 0.053*	0.069
Nigeria	− 0.119***	0.107	− 0.097***	0.136	− 0.141**	0.181
Uganda	− 0.162***	0.094	− 0.205***	0.131	− 0.109**	0.139
Constant	− 1.620	1.065	0.186	1.373	− 4.120**	1.785

*Source* Survey data (2021)

***, **, and * denote statistical significance at 1%, 5%, 10%, respectively

Holistically, these figures are lower than the household values in other studies (Akuffo and Quagrainie 2019; Tuholske et al. 2020), suggesting that food insecurity is higher among young people compared to taking the household as a unit. This further explains why measuring food consumption at the individual level is relevant against the regular household approach. The approximately 52% found outside the acceptable food consumption category, despite living above the poverty line as indicated in the income data, implies that income may not be a strong proxy for food security as hypothesized by some studies (Dunga 2020) since increased income may be directed to other needs such as purchasing assets, instead of food. This, however, necessitates concerted efforts towards improving individual food security status, particularly that of neglected vulnerable groups such as youths.

### Determinants of food security among young farmers

The determinants of food security among young farmers are presented in Table 4. The data was aggregated for ease of analysis and interpretation. Also, for better insights into gender disparities, the analysis was disaggregated by gender, identifying common and different factors that affect each gender group.

The result of the Hosmer and Lemeshow chi-square test conducted to test for the model’s goodness of fit suggests that the model is well-fitted since the p-value was insignificant and greater than 0.05 (*p*-value = 0.5491). Also, the test for multicollinearity shows no strong correlation between the variables included in the model. In addition, the c-statistics from the ROC curve was over 0.7 (Appendix 2), indicating that the model has predictive power. Finally, out of the fifteen explanatory variables included in the model, nine were found to be significant in determining food security for the pooled sample. These variables include access to extension service, credit, participation in the ENABLE-TAAT programme, having employees, access to the market, asset ownership, and land size.

For the gender-disaggregated analysis, four variables, including extension services, credit, participation in the ENABLE TAAT business incubation programme, and market access were significant for males while ten variables, including those that were significant for the pooled sample with the addition of age, farm income, and the Covid-19 variable were significant for female. The marginal effects of each explanatory variable are presented and discussed in this section.

Access to extension services positively and significantly (at *p* < 0.01) influenced the likelihood that a respondent would have an acceptable FCS. The value of the marginal effect implies that, for the pooled sample, having access to extension services increases the probability of being food secure by 16%. This could be attributed to the role of extension in linking young farmers to innovative opportunities that could help them profitably and sustainably run their enterprises. Access to such opportunities could also have a spill-over effect on their productivity. Similar results were obtained for both the male and female groups. Among the male and female respondents, access to extension services increased the probability of being food secure by 15% and 17%, respectively. The larger effect obtained for female respondents has insightful extension-targeting implications since they are the home caregiver and tend to be more concerned about food security issues than men. These results align with previous studies (Pan et al. 2018; Tesfaye et al. 2008; Yusuf et al. 2015), except for Ragasa et al. (2019), who found similar results for the pooled and male sample but an insignificant effect for the female group.

Access to credit was negatively and significantly (at *p* < 0.01) associated with food security, implying that those who had borrowed money in the last 12 months are less likely to have an acceptable food consumption score and, subsequently, likely to be more food insecure. Even though this is not expected since credit is expected to raise production and contradicts some literature where credit support is highlighted as a crucial aspect of promoting household food security (Aidoo et al. 2013; Iftikhar and Mahmood 2017; Matchaya and Chilonda 2012), the results could be attributed to the lack of creditworthiness among young farmers, who would most likely explore informal credit sources with high-interest rates and ridiculous repayment conditions. This also corroborates Ngema et al. (2018), who argued that higher loan repayment rates may necessitate households/individuals to lower consumption. However, our result corroborates Acheampong et al. (2022) who explained that farmers who have access to credit may not spend on food consumption and farming activities but, use it for other pressing issues such as purchasing assets, seeking health care, etc. Accessing this from a gendered perspective increased access to credit; otherwise, borrowing reduced the consumption scores of both male and female respondents. Participation in agribusiness empowerment programmes such as ENABLE-TAAT was found to positively and significantly (*p* < 0.01) influence food security by 11%. Similar results were obtained in the other two contexts considered (gendered-perspective). This suggests that participation in empowerment programmes increase the likelihood that a respondent, either male or female, will have an acceptable FCS. This could be attributed to the technical support and continuous mentorship from the programme and its focus on improving the food security status of young farmers through skills development and capacity building. This corroborates Garbero and Jäckering (2021), who found that agricultural programmes improve the food security status of beneficiaries, especially for those residing in food-insecure countries.

Contrary to prior expectations, having employees had a negative but significant influence on food security. The value and direction of the marginal effect imply that having employees reduce the likelihood of having an acceptable FCS by 6%. Even though more labour/employees are expected to raise output and productivity, the negative influence could be attributed to the additional cost incurred on wages and salaries. In cases where an increased number of employees do not translate into increased output or income, young farmers could experience adverse economic outcomes which may influence their food consumption. Alternatively, this may also be attributed to farmers' scale of operation. While this factor was insignificant for the male category, a similar result was obtained for the female group. Yusuf et al. (2015) also found similar results among urban farming households in Nigeria.

Following the marginal effects, access to the market increases the probability of being food secure by 20% for the pooled, male, and female samples, respectively. Access to market information, particularly the input market, may aid the adoption of improved inputs and better services that could contribute to increased production and income. This corroborates Ogunniyi et al. (2021) and Tesfamariam et al. (2018), who attributed improved food security to the positive effect of market information. This, however, contradicts Usman and Callo-Concha (2021), who found no significant relationship between household food security and market access, instead, market access encouraged smallholder households to rely less on their own production to improve household consumption diversity.

As expected, food security was negatively impacted by the Covid-19 pandemic. According to the results, the pandemic reduced the likelihood of being food secure by 7% and 12%, respectively for the pooled and female groups. This is in line with several studies that have discussed the negative effect of the pandemic on farming households. The significance of this variable suggests that female respondents were more affected by the Covid-19 restrictions than their male counterparts since it was insignificant for both the pooled and male samples.

With regards to location, the results showed that food insecurity is negatively and significantly associated with all the countries with a higher likelihood among respondents in Uganda (16% higher likelihood). This is not surprising as young Africans are more susceptible to food insecurity. Similar results were obtained for the three groups (pooled, female, and male) considered. This implies that, regardless of their location, young male and female African farmers are faced with the challenge of food insecurity.

Asides from the ones discussed so far, the analysis showed that one other factor determined food consumption among the female group. Age was positive and significant at *p* < 0.0 5, indicating that older youths are more likely to be food secure. The marginal effect suggests that an increase in age by 1 year led to a 22% increase in the log-odd of being food secure.

### Study limitations

Even though our study filled an important gap in the literature and is one of the few to provide empirical evidence on the level and determinants of food insecurity among young farmers in Kenya, Nigeria, and Uganda, the results should be interpreted in view of the following limitations. First, our sample may not be representative of young farmers in the three study countries. Thus, results generalization to the broader young farming population in and across these countries is limited and should be interpreted considering the original programme’s design. Nonetheless, our sample included young farmers in rural areas, which is more relevant to our study objectives. Second, even though we reviewed a few existing literature to identify relevant factors influencing food security, the list of determining factors included in the model is not exhaustive. Regardless of these limitations, our study contributes to the growing body of research on youth food security in cross-national settings. Future research should, thus, address these limitations for more insight into food (in)security among young farmers to inform how best to address its challenges following a youth-centered approach.

### Conclusions and policy recommendations

The study assessed food security and its determining factors among young African farmers. The assessment of Dietary Diversity showed that young farmers’ diets consisted mainly of cereals and root tubers and fats and oils across the study areas. Fruits and pulses were the least consumed which could be attributed to high purchase costs since the few that consumed took from own production. These results suggest low dietary diversity among the young farmers and the need for youth sensitization on the importance of having a diversified diet. Food security, proxied by FCS revealed that the majority of the young farmers are within the unacceptable food consumption group, defined by poor (13%) and borderline (38%) food consumption groups. This substantial percentage within the unacceptable group is quite worrying and suggests that efforts should be intensified toward improving food security among young farmers. Also, the results showed a high level of food insecurity among the Ugandan respondents. The odds of being food secure was positively determined by access to extension services, participation in the ENABLE TAAT business incubation programme, and access to market information but, negatively by access to credit, number of employees, the Covid-19 pandemic, and the location variables. In addition to these variables, the gender-disaggregated analysis showed that the food security status of the female group was positively influenced by age, suggesting that younger female youths are more likely to be food insecure.

These findings highlight the relevance of extension services, agribusiness empowerment programmes (ENABLE-TAAT), and access to market information in boosting the food security status of young farmers. Extension services link young farmers to improved agricultural practices and other innovative opportunities along the agricultural value chain, that can help to raise food production and improve food security. Also, these services can guide young farmers, particularly during the start-up stage. This, in turn, can reduce crop and agribusiness failure and contribute to food security.

Similarly, the findings show the importance of agribusiness incubation programmes such as the ENABLE-TAAT programme to the food security status of young farmers. One of the major challenges facing young farmers documented in literature is lack of relevant skills and mentorship. These programmes can help young farmers to build the much-needed agricultural skills and capabilities which could invariably improve their productivity. Thus, agribusiness empowerment programmes that focus on skills building, employment, and financial capability might be an appropriate intervention for young farmers. The significance of the ENABLE TAAT programme in improving food consumption across the three groups suggests the need to promote and facilitate young farmers’ participation in agribusiness incubation programmes. These results show that participation in agribusiness empowerment programmes is crucial to empowering young farmers to improve their skills and productivity, which is likely to impact their level of food consumption and, subsequently, food security status. To scale the impact of such programmes, participants could be encouraged to peer with other non-participants for knowledge and skill transfer. Also, the programme could be established as a permanent incubation center against the current 3-years plan. Concerning the effect of COVID-19, more efforts should target female farmers in coping with the impact of the pandemic and its undesirable outcomes.

The low percentage of respondents who had access to credit facilities and the negative effect of credit across the three groups suggest that young farmers may not be exploring the right credit channel from which they could generate positive outcomes. This calls for strategies to improve the creditworthiness of young farmers to aid access to the right and less-restrictive channels so that access could translate into better economic outcomes. An example is group borrowing which was effective among young business owners in the United States. However, the relationship between credit access and youth food security status should be further studied to better understand the dynamics of this issue.

## Figures and Tables

**Fig. 3:**
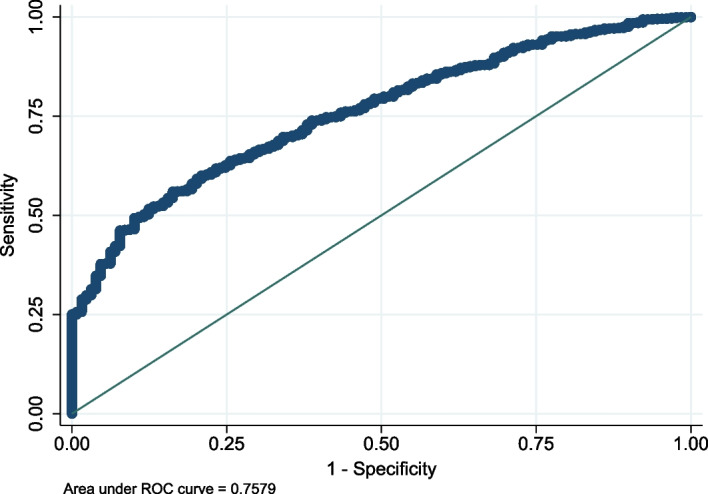
ROC curve

## Data Availability

The dataset used is available upon reasonable request from the authors.

## Appendix 1

See Table 5.

**Table 5:** Definition of variables included in the logit model. *Source* Authors compilation

Variables	Measurement	Hypothesized direction	References
Extension service	Dichotomous, taking the value 1 if a respondent has access to extension service and 0 otherwise	+	Pan et al. (2018), Tesfaye et al. (2008), Yusuf et al. (2015)
Gender	Respondent’s gender; 1 = male, 0 = female (Dummy)	+/−	Acheampong et al. (2022)
Credit	Measures whether an individual has access to credit or not; 1 = yes, 0 = no (Dummy)	+	Aidoo et al. (2013), Iftikhar and Mahmood (2017), Matchaya and Chilonda (2012)
Age	Age of respondents in years (Continuous)	+/−	Acheampong et al. (2022), Masa et al. (2020), Oyetunde-Usman and Olagunju (2019)
Education	Years of formal education of respondent (Continuous)	+	Acheampong et al. (2022), Matavel et al. (2022)
Household size	Number of household members (Continuous)	−	Masa et al. (2020), Matavel et al. (2022)
Residence	Current residence of respondent; 1=rural, 2=metropolitan, 3=Urban (Categorical)	−	Acheampong et al. (2022)
Ownership	Type of business ownership; 1=Sole owner, 0= Partnership (Dummy)	+/−	
ENABLE TAAT programme	Participation in the ENABLE TAAT programme; 1=participants, 0= Non-participants (Dummy)	+	Masa et al. (2020)
Employees	Number of employees (Continuous)	+	Inegbedion (2020)
Market information	Access to market information; 1=Have access, 0=Otherwise (Dummy)	+	Ogunniyi et al. (2021), Tesfamariam et al. (2018)
Asset ownership	Whether a respondent has agribusiness asset or not; 1=Yes, 0=No	+	Sisha (2020), Gebru et al. (2019)
Covid19	Whether agribusiness performance was affected by Covid19 or not; 1=Yes, 0=No	−	Workie et al. (2020)
Land size	Size of farmland owned in Hectares (Continuous)	−	Sisha (2020)
Farm Income	Total farm income (Continuous)	+	Acheampong et al. (2022), Matavel et al. (2022)

## Appendix 2

See Fig. 3.

## Footnotes

1 The ENABLE-TAAT programme was implemented across 7 African countries with the aim of involving youth in the process of agricultural transformation and improving their capacities in different agricultural value chains. Another key objective was to improve youths’ food security status and nutrition by promoting better practices and adoption of improved crop varieties among young farmers.
